# The diagnostic value of non-contrast enhanced quiescent interval single shot (QISS) magnetic resonance angiography at 3T for lower extremity peripheral arterial disease, in comparison to CT angiography

**DOI:** 10.1186/s12968-016-0294-6

**Published:** 2016-10-31

**Authors:** Gang Wu, Jun Yang, Tianjing Zhang, John N. Morelli, Shivraman Giri, Xiaoming Li, Wenlin Tang

**Affiliations:** 1 Department of Radiology, Tongji Hospital, Tongji Medical College, Huazhong University of Science and Technology, Wuhan, Hubei China; 2 Department of Vascular Surgery, Tongji Hospital, Tongji Medical College, Huazhong University of Science and Technology, Wuhan, Hubei China; 3 Northeast Asia Collaboration, Siemens Healthineers, Beijing, China; 4 St John Medical Center, Tulsa, OK USA; 5 Cardiovascular MR R&D, Siemens Healthcare, Chicago, USA; 6 MR Scientific, SIEMENS Healthcare, Shanghai, China

**Keywords:** Non-contrast enhanced, Magnetic resonance angiography, Lower extremity, Peripheral arterial disease

## Abstract

**Background:**

The high incidence of renal insufficiency in patients with Peripheral Arterial Disease raises the concern for nephrogenic systemic fibrosis (NSF) with respect to contrast enhanced MRA. The risk of NSF is eliminated with non-contrast enhanced magnetic resonance angiography. The purpose of the current study is to compare image quality and diagnostic performance of non-contrast enhanced Quiescent Interval Single Shot (QISS) magnetic resonance angiography at 3 T versus CT angiography for evaluation of lower extremity Peripheral Arterial Disease (PAD).

**Methods:**

32 consecutive patients (23 male, 9 female, age range 40–81 years, average age 61.97 years) with clinically suspected lower extremity PAD underwent QISS MRA and CTA. 19 of 32 patients underwent Digital Subtraction Angiography (DSA). Image quality of MRA was compared with CTA by two radiologists with 10 and 8 years’ experience according to a 4-point scale. The Kappa test was used to determine the intermodality agreement between MRA and CTA in stenosis assessment, and interobserver agreement with each method. Sensitivity and specificity of CTA and MRA in detecting hemodynamically significant stenosis (≥50 %) were compared, with DSA serving as reference standard when available.

**Results:**

Image quality of QISS MRA was rated 3.70 ± 0.49 by reader 1, and 3.72 ± 0.47 by reader 2, significantly lower than that of CTA (3.80 ± 0.44 and 3.82 ± 0.42, *P* < 0.001 for both readers). Intermodality agreement between MRA and CTA was excellent for assessment of stenosis (Kappa = 0.923 ± 0.013 for reader 1, 0.930 ± 0.012 for reader 2). Interobserver agreement was 0.936 ± 0.012 for CTA and 0.935 ± 0.011 for MRA. For readers 1 and 2 respectively, the sensitivity of QISS was 94.25 and 93.26 % (versus 90.11 and 89.13 % for CTA, *P* > 0.05), and specificity of QISS was 96.70 and 97.75 % (versus 96.55 and 96.51 % for CTA, *P* > 0.05). For heavily calcified segments, sensitivity of QISS (95.83 and 95.83 %) was significantly higher than that of CTA (74.19 and 76.67 %, *P* < 0.05).

**Conclusion:**

QISS is a reliable alternative to CTA for evaluation of lower extremity PAD, and may be suitable as a first-line screening examination in patients with contraindications to intravenous contrast administration.

## Background

Peripheral arterial disease (PAD) affects more than 5 million adults in the United States [1], and the morbidity associated with PAD is increasing duo to the aging population. Accurate diagnosis of PAD is essential for treatment and management [2]. Ankle brachial index (ABI) measurement is a sensitive test and is often the first line test performed to evaluated PAD [3]. Color Doppler ultrasonography (CDU) is widely utilized in patients with chronic symptomatic limb ischemia, as it is a safe, noninvasive, and inexpensive examination [4]. However, CDU does not fully depict the entirety of the lower extremity vasculature and is operator dependent. Digital Subtraction Angiography (DSA) is considered the clinical gold standard for the diagnosis of PAD due to its high spatial and temporal resolution [5]. In addition, treatment of significant stenoses with balloon angioplasty and stenting can be performed concurrently. However, DSA is utilized relatively infrequently in practice as it is an invasive and expensive test and relies upon the use of ionizing radiation. Computed tomography angiography (CTA) and magnetic resonance angiography (MRA) are non-invasive alternatives to DSA for the depiction of PAD [6, 7]. The diagnostic performance of CTA and contrast-enhanced magnetic resonance angiography (CE MRA) have been reported as comparable to that of DSA in many studies [8, 9]. However, the high incidence of renal insufficiency in patients with PAD raises the concern for nephrogenic systemic fibrosis (NSF) with respect to contrast enhanced MRA [10, 11]. The risk of NSF is eliminated with non-contrast enhanced magnetic resonance angiography (NCE-MRA) techniques which have been developed as alternatives to CE-MRA [12–14]. The Quiescent Interval Single-shot (QISS) technique was developed as a safe and simple “push-button” NCE MRA technique. It uses saturation pulses to suppress background and venous signal, and is ECG-gated to synchronize data acquisition with maximal arterial inflow. The use of a single-shot 2D balanced Steady State Free Precession (b-SSFP) sequence results in clear depiction of arteries. The diagnostic performance of QISS MRA has been reported to be comparable to that of CE MRA [15]. To the best of our knowledge, there is no study that has compared QISS at 3 T with CTA. Imaging at 3 T is an attractive option due to the increased SNR and the possibility to use higher parallel imaging acceleration factors. The aim of the current study is to compare non-contrast enhanced QISS MRA at 3 T with CTA for the evaluation of lower extremity arterial disease.

## Methods

### Patients

This prospective study was approved by the local institutional review board. Inclusion criteria were: (1) patients with symptomatic lower extremity ischemia and (2) patients who agreed to both CTA and non-contrast enhanced QISS MRA. Exclusion criteria were: (1) patients with renal insufficiency (GFR < 30 ml/min/1.73 m^2)^ in whom CT contrast was contradicted and (2) patients with contraindications to MR (i.e. A pacemaker or claustrophobia). From December 2014 to July 2015, 32 patients (23 men, 9 women, age range 40–81 years, average age 61.97 years) met the inclusion criteria and were enrolled in this study. Main symptoms of the patients were limb pain and claudication, with an average duration of 11.5 months. Duration was less than 3 months for 5 patients, 3 ~ 12 months for 20 patients, and more than 12 months for 7 patients. Creatinine level was from 41 to 228 μmol/L, with an average of 76.3 μmol/L. Glomerular Filtration Rate (GFR) was from 34.5 to 149 ml/min/1.73 m^2^, with an average of 84.96 ml/min/1.73 m^2^. GFR was less than 60 ml/min/1.73 m^2^ for 7 patients. Main pertinent medical history was smoking (*n* = 14), diabetes (*n* = 19), hypertension (*n* = 17) and coronary heart disease (*n* = 6). Written informed consent was obtained from all patients before the examinations. QISS MRA and CTA examinations were performed on the same day. QISS MRA was performed prior to CTA in 28 cases, and after CTA in 4 cases. 19 of the 32 patients underwent DSA for definitive diagnosis or treatment within two days following CTA and MRA.

### MR angiography

All non-contrast enhanced QISS MRA examinations were performed on a 3.0 T whole-body MR system (MAGNETOM Skyra, Siemens Healthcare, Erlangen, Germany). Patients were placed on the scanner in feet-first supine position. A dedicated peripheral coil and two eight-element body array coils were used to cover the lower extremity and lower abdomen, and were combined with the posterior integrated multi-channel spine coil. Electrocardiographic triggering was used to ensure proper synchronization between the arterial inflow events and data sampling. Initially a scout image (Fast_View_Scout) was performed of the whole lower extremity and abdomen for localization purposes using the following parameters: TR/TE, 2.56/1.44 ms; FOV, 48 cm × 149 cm; slice thickness, 5 mm. QISS MRA was performed in the transverse plane with the following parameters: TR = 1 heart beat; TE = 1.68 ms; flip angle, 90, or reduced according to SAR limitation; bandwidth, 700Hz; FOV, 400 mm × 260 mm; matrix, 400 × 261; number of slices, 40; slice thickness, 3 mm; GeneRalized Autocalibrating Partially Parallel Acquisition (GRAPPA) factor, 3 or 2. QISS was performed in 9 stations from the distal calf to the lower abdomen in order to cover the distal abdominal aorta. For the lower 7 stations, acquisition time was equal to 40 heart beats at each station or approximately 0.5 min for a heart rate of 80/min. For the upper two stations in the pelvis and lower abdomen, only 10 slices were imaged during a breath hold, followed by a rest interval of 8 s. The data acquisition was performed in approximately 6.5 min, given an average heart rate of 80/min. The total study time was lengthened by the shimming process, which was repeated at each station. Coronal Maximum Intensity Projection (MIP) images of each station were generated by the scanner software, and all the MIP images were automatically spliced into a composite image including the entire region of interest.

### CT angiography

All CTA examinations were performed at a 128-row CT scanner (Discovery HD 750, GE medical, America), with the following parameters: tube voltage, 100 Kv; tube current, 150 mA; pitch, 0.984:1; table speed, 55 mm/s; slice thickness, 0.625 mm; FOV, 50 cm. Iodinated contrast agent (Ultravist, Bayer, Germany, 1.2 ml/kg body weight) was administered via an electronic power injector (Stellant, MEDRAD, America) through an 18 gauge intravenous line placed in the right cubital vein, at a rate of 3 ml/s. The bolus-tracking technique was used whereby a region of interest (ROI) was positioned at the aortic bifurcation. Image acquisition automatically started 5.5 s after the attenuation in the ROI reached the predefined threshold of 120 Hounsfield Units (HU).

### Digital subtraction angiography

19 of 32 patients underwent DSA performed by an interventional radiologist with 15 years’ experience on a clinical DSA unit (Allura Xper FD20, Philips Healthcare, The Netherlands). 6 mL of iodinated contrast material (Ultravist, Bayer, Germany) were administered intra-arterially at a rate of 3 ml/s for each DSA run. 18 patients received balloon angioplasty duo to a severe stenosis or occlusion. Stents were placed in 5 cases. A patient with thrombosis occluding the left femoral artery received arterial embolectomy.

### Data analysis

Post-processing procedures and measurement were performed on a dedicated Siemens workstation (Syngo. Via, Siemens Healthcare, Erlangen, Germany). CTA MIP images were reconstructed with a window setting of 600/300 (window width/window level). Two readers with 10 and 8 years’ experience graded the image quality for each segment using source images as well as reconstructed images (MIP, and multi-planar reconstruction). Image quality of the following 19 segments was evaluated separately: 1, distal abdominal aorta; 2/3, common iliac artery; 4/5, external iliac artery; 6/7, internal iliac artery; 8/9, femoral artery; 10/11, femoral profound artery; 12/13, popliteal artery; 14/15, anterior tibial artery; 16/17, posterior tibial artery; 18/19, peroneal artery. The readers blinded to clinical information evaluated segments in random order, using a 4-point scale: 1 = poor or nondiagnostic arterial display; 2 = fair arterial display and delineation of the arterial structures with detection of lesions still possible; 3 = good arterial display without impaired delineation of the vascular structures; 4 = excellent arterial display with sharp delineation of the arteries throughout their length.

The arterial stenosis severity was rated by two readers with 11 years’ and 9 years’ experience respectively. Blinded readers were allowed to use both source images and reconstructed images (including MIP, and multi-planar reconstruction) for stenosis evaluation. A grading system proposed by the American College of Radiology in a multi-institutional trial of peripheral MRA was used for arterial stenosis evaluation [16]: 0, normal; 1, minimal stenosis of less than 50 %; 2, one lesion with 50 % or greater stenosis; 3, more than one lesion with 50 % or greater stenosis; 4, occlusion. Each segment at QISS MRA was assigned a score. Evaluation with CTA was performed using the same criteria as with MRA. Intermodality agreement and interobserver agreement for stenosis rating was determined on a per segment basis.

For the 19 patients with DSA examination, the segments at DSA and corresponding segments at MRA and CTA were evaluated by two readers with 10 years’ and 11 years’ experience for the presence of significant stenosis (≥50 %). If multiple stenoses were found in a segment, the most severe stenosis was analyzed. One reader evaluated QISS MRA segments first, then CTA. The other reader evaluated CTA segments first, then QISS. The interval between evaluations was 4 weeks to avoid recall bias. Sensitivity, specificity, and accuracy of QISS and CTA for detection of significant stenosis (≥50 %) were calculated on a segment basis with DSA serving as the reference standard.

### Statistical analysis

All data analysis was performed using SPSS (version 21.0, IBM, America). Image quality was compared between CTA and QISS MRA using a Wilcoxon signed rank test. The intermodality agreement between MRA and CTA and interobserver agreement with each method in assessment of arterial stenosis was determined with a Kappa test. Kappa > 0.8 was considered as excellent agreement. 0.6–0.8 was considered good. 0.4–0.59 was considered fair. Kappa < 0.4 was considered as poor agreement. The sensitivity, specificity and accuracy were calculated with DSA serving as the reference standard, and were compared between MRA and CTA using a Spearman chi-square test. *P*-values less than 0.05 were considered statistically significant.

## Results

**Fig. 1:**
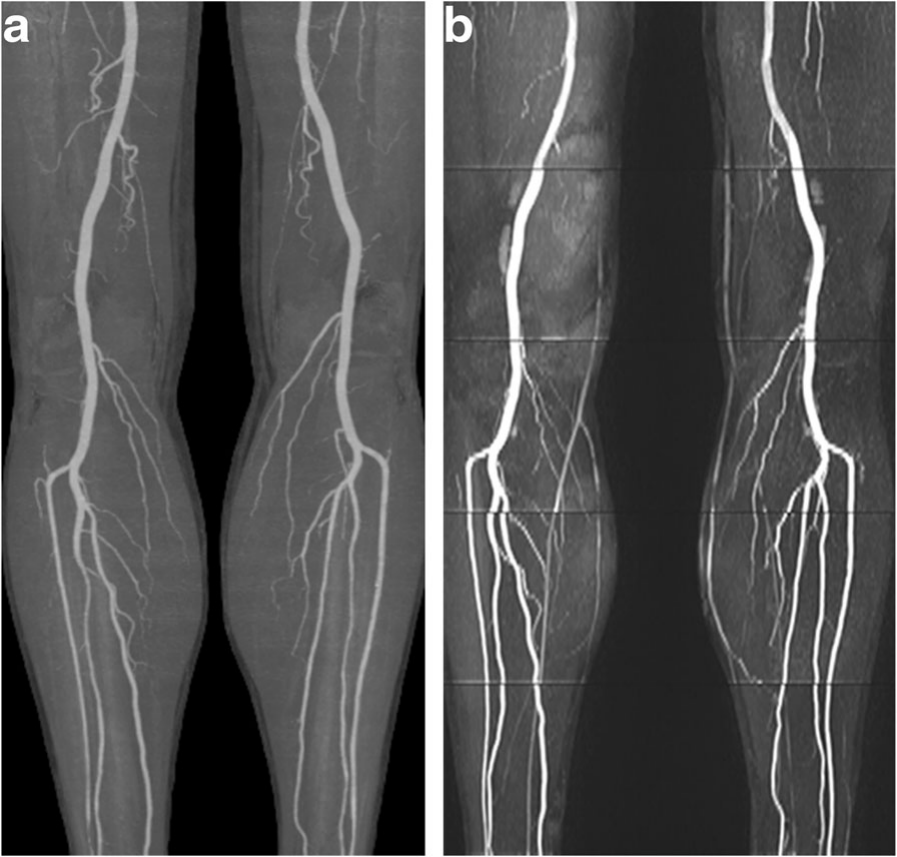
**a** Segments including popliteal artery, anterior tibial artery, posterior tibial artery and peroneal artery were rated as excellent in quality with CTA. **b** These segments were rated as excellent in quality with QISS MRA

All 32 patients completed the CTA and MRA examinations successfully, without any adverse events. With 19 lower extremity arterial segments assessed per patient, a total of 608 segments were evaluated. Segments with nondiagnostic image quality (score 1) were not observed with QISS MRA or CTA in the present study. 3.78 % (23/608) MRA segments were rated as fair in quality (score 2) by Reader1, and 3.62 % (22/608) by Reader 2. 77.96 % (474/608) MRA segments were rated as excellent in quality (see Fig. 1) by Reader 1, and 78.29 % (476/608) by Reader 2. 0.66 % (4/608) CTA segments were rated as fair in quality (score 2) by Reader 1, and 0.33 % (2/608) by Reader 2. 92.27 % (561/608) CTA segments were rated as excellent in quality (score 4) by Reader1, and 94.08 % (572/608) by Reader 2. Total image quality of CTA was significantly higher than that of QISS MRA (*P* < 0.001 for both readers, see Table 1). Notably, the image quality of the two methods was not statistically significantly different at some segments (*P* > 0.05 for both readers, see Table 1). Of the 608 segments evaluated, 77 were heavily calcified segments. For these segments, image quality of QISS were significantly higher than that of CTA (*P* < 0.001 for both readers, see Table 1).

**Table 1:** Image quality was compared between CTA and MRA. 1 = poor or nondiagnostic arterial display; 2 = fair arterial display and delineation of the arterial structures with detection of lesions still possible; 3 = good arterial display without impaired delineation of the vessel structures; 4 = excellent arterial display with sharp delineation of the arteries throughout their length

	Reader 1			Reader 2		
	CTA	MRA	P	CTA	MRA	P
Distal abdominal aorta	3.91 ± 0.30	3.72 ± 0.46	0.034	3.94 ± 0.25	3.63 ± 0.49	0.002
Common iliac artery	3.86 ± 0.43	3.61 ± 0.55	0.003	3.88 ± 0.38	3.66 ± 0.48	0.002
External iliac artey	3.81 ± 0.50	3.55 ± 0.53	0.005	3.86 ± 0.39	3.64 ± 0.52	0.002
Internal iliac artery	3.63 ± 0.55	3.58 ± 0.56	0.554	3.64 ± 0.55	3.64 ± 0.48	0.981
Femoral artery	3.78 ± 0.49	3.67 ± 0.47	0.217	3.78 ± 0.55	3.61 ± 0.52	0.081
Femoral profound artery	3.97 ± 0.18	3.75 ± 0.44	<0.001	3.94 ± 0.24	3.77 ± 0.43	0.002
Popliteal artery	3.84 ± 0.41	3.75 ± 0.53	0.206	3.86 ± 0.39	3.78 ± 0.45	0.225
Anterior tibial artery	3.70 ± 0.46	3.78 ± 0.45	0.251	3.73 ± 0.45	3.80 ± 0.44	0.433
Posterior tibial artery	3.73 ± 0.45	3.80 ± 0.44	0.371	3.78 ± 0.42	3.80 ± 0.44	0.827
Peroneal artery	3.78 ± 0.42	3.81 ± 0.39	0.593	3.81 ± 0.43	3.84 ± 0.37	0.655
Total	3.80 ± 0.44	3.70 ± 0.49	<0.001	3.82 ± 0.42	3.72 ± 0.47	<0.001
Heavily calcified segment	3.13 ± 0.77	3.68 ± 0.59	<0.001	3.22 ± 0.68	3.65 ± 0.60	<0.001

*CTA* computed tomography angiography, *MRA* magnetic resonance angiography

15 of fair (score 2) segments at QISS were caused by limb motion. In the pelvic region of 3 patients, vessel blurring and misregistration due to respiratory artifacts was observed. For one patient with orthopedic implant at the right femur, image quality degradation of CTA was not caused, while the QISS segment neighbor to fixator (right femoral artery) was rated as fair (score 2). Image degradation caused by the implant was not observed at other segments of this patient. For 3 patients with cardiac arrhythmia, QISS examination time was lengthened, because data acquisition was not performed every heart beat. Significant artifacts or image degradation was not observed.

**Fig. 2:**
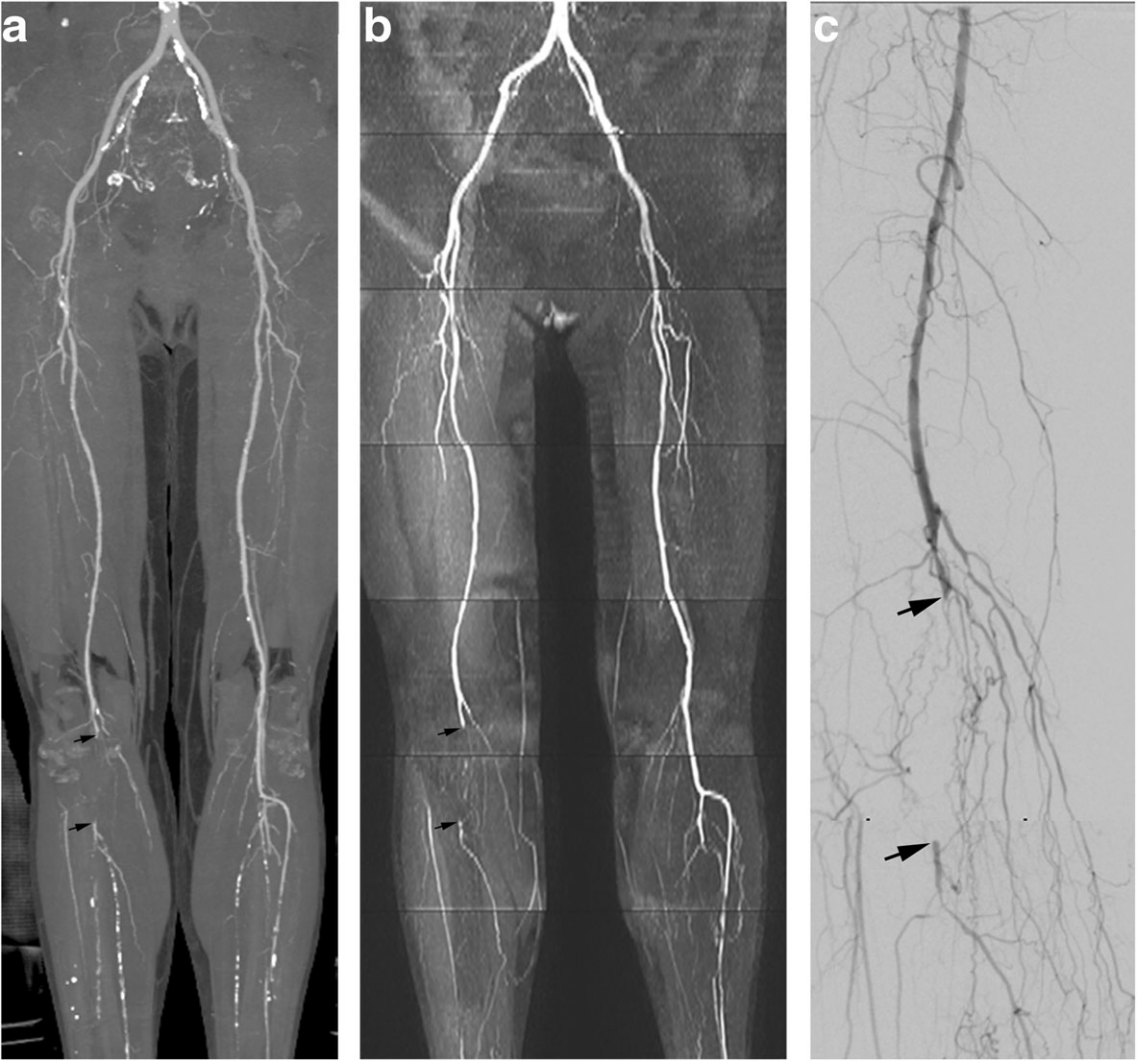
**a** Multiple stenoses at right femoral artery and occlusion (*arrows*) of right popliteal artery were shown with CTA image; however, occlusions at bilateral lower leg were difficult to identify duo to multiple calcified plaques. **b** Occlusion of the right popliteal artery was also shown with QISS (*arrows*). Calcified plaques were not problematic with QISS, with occlusions easily identified at calf. **c** Occlusion of the right popliteal artery was proved by DSA (*arrows*). More collateral circulation vessels were shown with DSA

Intermodality agreement in stenosis ratings was calculated on a per segment basis. Stenosis ratings were equivalent with the two methods (Fig. 2) in 94.24 % segments (573/608) for Reader 1, and 94.74 % (576/608) for Reader 2. Intermodality agreement was excellent for rating stenoses (kappa > 0.8, for both readers, see Table 2). Interobserver agreement was excellent for both CTA (0.936 ± 0.012) and MRA (0.935 ± 0.011).

**Table 2:** Intermodality agreement between CTA and MRA in rating stenosis. The arterial stenosis degree: 0, normal; 1, minimal stenosis of less than 50 %; 2, one lesion with 50 % or greater stenosis; 3, more than one lesion with 50 % or greater stenosis; 4, occlusion

Reader 1							Reader 2					
		MRA						MRA				
		0	1	2	3	4		0	1	2	3	4
CTA	0	217	11	0	0	0	0	216	11	0	0	0
	1	4	150	3	1	0	1	3	152	2	1	0
	2	0	5	43	2	0	2	0	5	45	2	0
	3	0	2	4	74	1	3	0	1	5	74	0
	4	0	0	0	2	89	4	0	0	0	2	89
kappa	0.923 ± 0.013						0.930 ± 0.012					

*CTA* computed tomography angiography, *MRA* magnetic resonance angiography

**Fig. 3:**
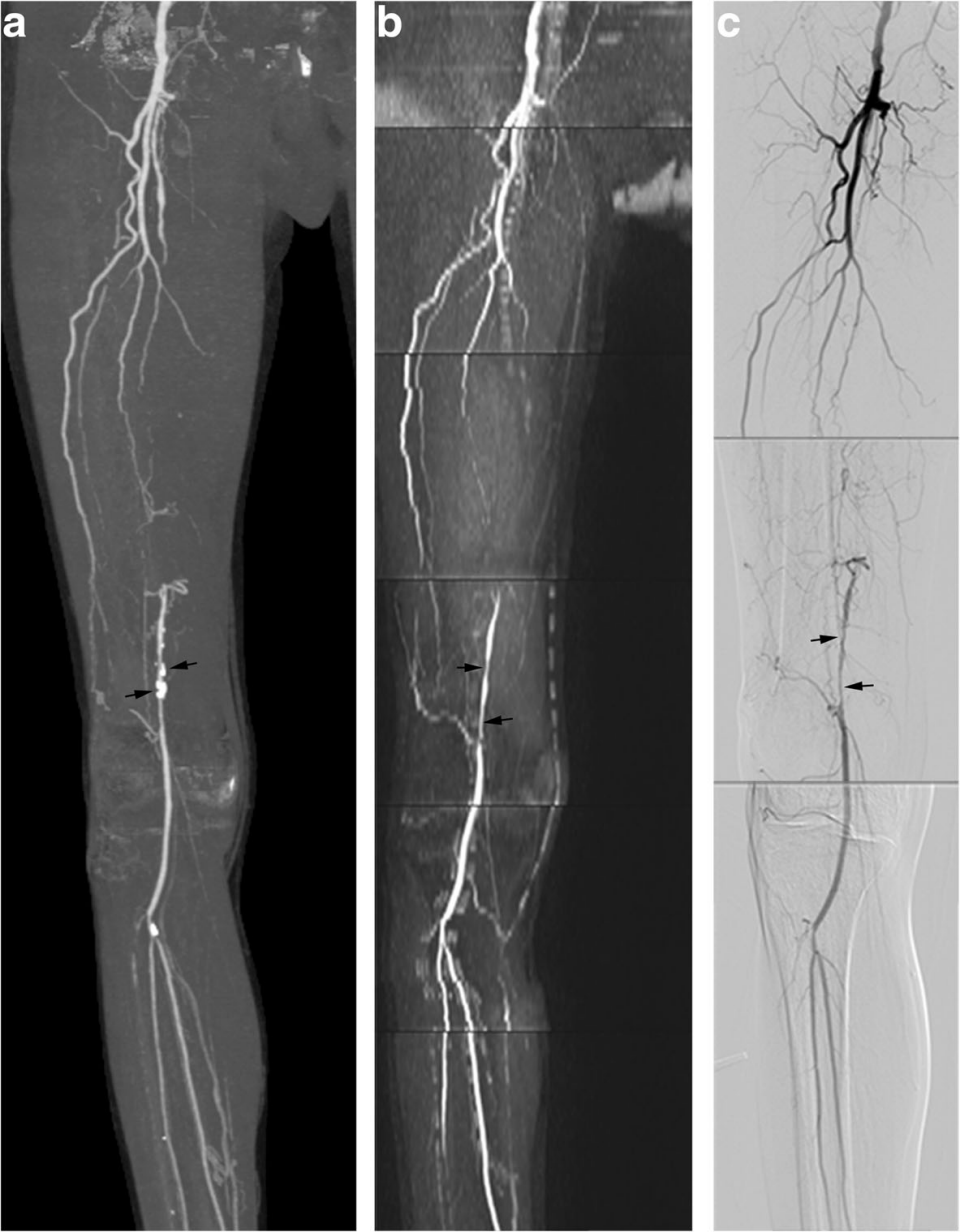
Occlusion of right femoral artery was seen at CTA (**a**) and QISS MRA (**b**), and was proved by DSA (**c**). Stenosis degree is difficult to determine duo to calcified plaques overlapping at CTA MIP image (*arrows*). Significant stenoses (*arrows*) were well seen at QISS MIP image (**b**) and DSA (**c**)

In the present study DSA was performed on the segments where severe vascular disease was identified by CTA/MRA. In total, 178 segments were evaluated with DSA. Significant stenoses (≥50 %) were found in 85 segments (Fig. 3). Using DSA as the reference standard, sensitivity of QISS MRA was 94.25 and 93.26 %, and specificity was 96.70 and 97.75 %. Sensitivity, specificity and accuracy of QISS MRA for the detection of significant stenosis (≥50 %) were not statistically significantly different from those of CTA (see Table 3). Of 178 segments evaluated with DSA, 41 segments were heavily calcified. For these segments, sensitivity of QISS in detecting significant stenoses (≥50 %) was significantly higher than that of CTA (see Table 3).

**Table 3:** Sensitivity, specificity and accuracy of CTA and MRA in detecting significant stenoses (≥50 %) using DSA as the reference standard

	Reader 1			Reader 2		
	CTA	MRA	P	CTA	MRA	P
All segments						
Sensitivity	90.11 % (82/91)	94.25 % (82/87)	0.305	89.13 % (82/92)	93.26 % (83/89)	0.328
Specificity	96.55 % (84/87)	96.70 % (88/91)	0.955	96.51 % (83/86)	97.75 % (87/89)	0.622
Accuracy	93.26 % (166/178)	95.51 % (170/178)	0.357	92.70 % (165/178)	95.51 % (170/178)	0.261
Heavily calcified segments						
Sensitivity	74.19 % (23/31)	95.83 % (23/24)	0.031	76.67 % (23/30)	95.83 % (23/24)	0.049
Specificity	90.00 % (9/10)	94.12 % (16/17)	0.693	90.91 % (10/11)	94.12 % (16/17)	0.747
Accuracy	78.05 % (32/41)	95.12 % (39/41)	0.023	80.49 % (33/41)	95.12 % (39/41)	0.043

*CTA* computed tomography angiography, *MRA* magnetic resonance angiography

## Discussion

Given the high incidence of renal insufficiency in PAD patients, the risk of contrast-induced nephropathy with CTA and MRA are important concerns [17, 18]. In such patients, non-contrast enhanced MRA is a promising alternative. Various NCE MRA technologies have been studied; however, none of them have been widely adopted in clinical practice duo to various difficulties. For example, 2D TOF has fallen into disuse because of lengthy imaging time and poor image quality. Electrocardiographic gated 3D fast spin-echo sequences have been shown to enable accurate imaging of the calf and pedal arteries, but these techniques requires optimal selection of systolic trigger delays [19, 20]. Flow-sensitive dephasing (FSD) prepared balanced steady state free precession sequence requires systolic and diastolic blood signal acquisition, and permits visualization of arteries with subtraction [21]. However, the FSD technique is not user-friendly as it requires the MR technologist to adjust multiple parameters from patient to patient [22]. QISS MRA is a bright blood sequential 2D NCE MRA technique originally developed for the evaluation of PAD [15]. Compared with other NCE MRA technologies, QISS has an easy “push-button” workflow, eliminating the need for extensive patient-to-patient parameter modification [23]. QISS has been compared with ABI in screening PAD [24], and has demonstrated a comparable diagnostic performance to CE-MRA for PAD evaluation [15, 25]. QISS has also been compared favorably to other NCE-MRA technique [26]. However, to the authors’ knowledge, QISS performance at 3 T has not been compared with CTA, the predominant test for PAD in many institutions because of its speed and accuracy. In the current study, QISS at 3 T was compared to CTA finding excellent intermodality agreement in the assessment of stenoses. For heavily calcified segments, the sensitivity of QISS in detecting significant stenoses (≥50 %) was significantly higher than that of CTA.

In the present study, the overall image quality of QISS MRA was lower than that of CTA, the difference being statistically significant. One possible explanation is that the spatial resolution of CTA (1 mm × 1 mm × 0.625 mm) is greater than that of QISS MRA (1 mm × 1 mm × 3 mm). Another possible explanation is that the acquisition time of CTA is much shorter than MRA, rendering it less prone to motion artifacts. However, it is worth noting that the image quality of the two methods was not significantly different at some segments.

Sufficiently suppressed background was observed in the source images of QISS MRA, resulting from the in-plane saturation pulse. The use of a gated acquisition with a b-SSFP sequence ensured high blood signal and adequate vessel-to-background contrast. Compared to other NCE-MRA techniques, QISS has superior flow contrast, because the thin-slice 2D QISS acquisition enables much greater replenishment of saturated arterial spins compared to other thick-slab 3D acquisitions. QISS is also relatively insensitive to patient motion due to the 2D acquisition in the transverse plane. NCE QISS MRA offers users the option of repeating acquisitions of arterial segments in cases of poor image quality due to motion or other technical limitations. For the current study no acquisitions were repeated due to imaging time considerations; however, this could be done in clinical practice to potentially further improve image quality.

The intrinsically high blood signal intensity with the b-SSFP sequence contributed to the image quality of QISS [27]. QISS MRA was also performed at 3 T where the intrinsic SNR is increased over lower field strengths [28, 29]. The assessment of stenoses with MRA and CTA was equivalent in most segments, and intermodality agreement was excellent. Thus, QISS MRA is a promising alternative to CTA, in particular because ionizing radiation and iodinated contrast are avoided.

2D QISS MRA with a slice thickness of 3 mm was utilized in the present study. A thinner slice thickness could be chosen; however, the number of slices required to cover the field of view would be increased with an associated increase in scan acquisition time. For example, the acquisition time doubles with slice thickness reduced from 3 mm to 1.5 mm. A lengthy acquisition poses difficulty for PAD patients with back or leg pain and the inability to keep still in supine position for extended periods. Fortunately, a 3 mm thickness was adequate for assessment of PAD in the present study, and the corresponding image acquisition time was acceptable (6.5 min for heart rate of 80 per minute).

QISS was performed in the transverse plane, similar to CTA. Because the scan direction was perpendicular to the lower extremity artery, they were sensitive to stenoses in both anterior-posterior and left-right directions, which may explain the excellent agreement in stenoses rating. In the future clinical practice, through-plane resolution could be further increased in a repeat-scan for a more detailed analysis of suspected segments.

The reported sensitivity of CTA for the detection of greater than 50 % stenosis is on the order of 89–100 % [30]. Stenosis degree may be overestimated when severely calcified antherosclerotic plaques were present [31]. Calcified plaque is not problematic with QISS MRA, likely accounting for the higher sensitivity of the technique. The diagnostic accuracy of QISS shown in the current study indicates that it may be a promising alternative to DSA.

The present study has several limitations. First, the sample size is small. More patients with lower extremity PAD should be included in the future studies. Second, DSA was available for only 19 patients. This reflects the fact that CTA currently performs sufficiently well in PAD patients to be considered the first-line examinations for most patients. Due to its invasiveness and cost, DSA was only performed on the patients with need of definitive diagnosis or endovascular therapy. Third, DSA was only performed on the diseased segments of the lower extremity arteries. Ionizing radiation and contrast administration would increase if all parts of lower extremity arterial vasculature were imaged. The number of segments evaluated by DSA was thus relatively small, weakening the statistical power. Fourth, the pedal arteries were not evaluated in the present study. Arteries of the foot are smaller in size and tortuous, thus making the image quality provided by a 2D acquisition inadequate for evaluation. However, accurate diagnosis for pedal PAD is as important as in the remainder of the lower extremities, especially in patients with diabetes [32]. Further advancement of NCE techniques will be required for sufficient evaluation of the pedal vasculature. Finally, a head-to-head comparison was not performed between QISS and other NCE MRA techniques as the focus was on comparison with CTA.

## Conclusions

In conclusion, the extent and severity of lower extremity PAD is accurately evaluated by NCE QISS MRA with a typical acquisition time of 7–9 min. QISS is a reliable alternative to CTA for this assessment, and may be suitable as a first-line screening examination for lower extremity PAD patients with contraindications to intravenous contrast administration.

## Abbreviations

CTA: Computed tomography angiography
DSA: Digital subtraction angiography
MRA: Magnetic resonance angiography
PAD: Peripheral arterial disease
QISS: Quiescent interval single shot
